# PREDICT *Prostate*, a useful tool in men with low- and intermediate-risk prostate cancer who are hesitant between conservative management and active treatment

**DOI:** 10.1186/s12916-020-01681-z

**Published:** 2020-07-16

**Authors:** Gaëtan Devos, Steven Joniau

**Affiliations:** Department of Urology, University Hospitals Leuven, Leuven, Belgium

**Keywords:** Prostate cancer, Prognosis, Prostate cancer-specific mortality, Decision aid

## Background

Risk stratification tools are useful to optimize treatment decisions in each individual patient, reducing both under- and overtreatment. Currently, several such tools are available to estimate disease aggressiveness in patients with localized prostate cancer (PCa). These include nomograms (e.g., Memorial Sloan-Kettering Cancer Center (MSKCC) nomogram), tiered classification systems (e.g., D’Amico, National Comprehensive Cancer Network (NCCN)), and risk assessment scoring systems (e.g., the cancer of the prostate risk assessment (CAPRA) score) [1]. Preoperative parameters such as biopsy Gleason score (or ISUP grade), initial PSA at time of biopsy, and clinical T-stage at digital rectal examination are used in these tools. The D’Amico classification, an easy-to-use three-tier classification system based on these three preoperative parameters, is adopted in daily clinical practice and endorsed by several international guidelines, such as the European Association of Urology (EAU) prostate cancer guidelines [2]. However, most of these risk stratification tools are designed to predict the risk of PSA recurrence after primary treatment rather than survival and do not take into account individual competing risk mortality in terms of age and comorbidity.

## PREDICT *Prostate*, a novel promising tool

The PREDICT *Prostate* tool, on the other hand, is a multivariable prognostic model that provides individualized cancer-specific and overall long-term survival estimates in localized PCa patients [3]. In addition to the use of routinely available preoperative clinical-pathological variables such as PSA, biopsy Gleason score (ISUP grade group), and clinical T-stage, the PREDICT *Prostate* tool also includes the impact of patient characteristics (age and comorbidity status) and radical treatment (radical prostatectomy or radiotherapy) on survival. Thurtle et al. [4] performed an external validation of their previously published PREDICT *Prostate* model. Applied to the large Swedish PCBaSe cohort, the tool was able to discriminate patients who faced PCa-specific mortality and outperformed other widely used models such as the CAPRA and the three-tier EAU classification. It was proven to generally have high c-indices for all-cause and PCa-specific mortality, and the model calibration was good and remained accurate within the treatment subgroups.

However, some issues need to be emphasized before using the tool in daily clinical practice. First, the two cohorts (original United Kingdom (UK) and Swedish PCBaSe cohort) are generally very similar in epidemiological characteristics. Yet, more than half (53%) of the patients in the PCBaSe had low-grade disease (ISUP grade group 1) compared to 32% in the original UK cohort. This high number of low-risk patients may affect the discriminatory power of the tool as these men are unlikely to die from their PCa. Second, although reasonably high numbers of high-risk PCa patients (according to EAU risk grouping system) were included in the development and PCBaSe cohorts (22.4% and 15.1% ISUP grade group 4–5 PCa, respectively; 14.5% and 16% stage T3–4, respectively), it is unclear how well the model performs in actively treated high-risk and very-high-risk PCa patients. International guidelines recommend conducting multimodal therapy in high-risk PCa patients combining surgery, radiation therapy, and systemic therapy [5]. However, multimodal therapy was not considered radical therapy in these cohorts. In addition, the model assumes “equality” of surgery and radiotherapy as radical therapy. While this may be true for low-risk and some intermediate-risk PCa patients [6], this is far from certain in high-risk, locally advanced disease. In addition, a large proportion of the patients were treated with androgen deprivation therapy (ADT) alone (31.5% in the original UK cohort and 23.1% in the external validation Swedish PCBaSe cohort). The PCa patients receiving ADT monotherapy tended to have high-risk localized PCa (71% and 78.8% in the original and PCBaSe cohort, respectively) or likely to have significant comorbidities excluding active local treatment. Approximately half of the high-risk patients in the original and external validation cohorts (47% and 52%, respectively) received ADT monotherapy. Today, international PCa guidelines strongly recommend against the use of ADT monotherapy in newly diagnosed, non-metastatic PCa patients [2]. Therefore, the model is less useful in optimizing the therapy decision in this high-risk population. Third, the type of risk stratification tools that have been used to compare the prognostic value of the PREDICT model (NCCN and EAU) are risk grouping systems and are not developed to predict mortality. On the other hand, it would be of great importance to compare, for example, the PREDICT *Prostate* model with the nomogram proposed by MSKCC, which is similar [7]. In addition, the PREDICT *Prostate* model does not contain genomic tests or molecular markers. The combination of clinical and genomic biomarkers’ tests (such as Decipher) has already been shown to improve the prognostic ability [8, 9]. Ideally, the model should be validated using data from prospective, randomized trials such as the PROTECT dataset [6]. Furthermore, the comorbidity status in PREDICT *Prostate* is defined by “any hospital admission in the last 2 years prior to PCa diagnosis.” While this allows clinicians to assess the impact of competing risks on the benefit of treatment, the implementation of more detailed comorbidity assessments such as the Charlson Comorbidity Index at the time of diagnosis will certainly improve the prognostic model [10]. Finally, the tool does not take into account recent changes in PCa management, such as the implementation of MRI prior to prostate biopsy or the use of targeted biopsies.

## Conclusions

Nevertheless, the results of the external validation are impressive and of great importance to the uro-oncological community to optimize treatment decisions in localized PCa patients. By including the impact of radical treatment on survival, the tool is especially useful in men who are hesitant between active surveillance or active treatment within their own context of competing mortality. However, the utility of the PREDICT *Prostate* tool in high-risk PCa patients is questionable.

## Data Availability

Not applicable

## Abbreviations

ADT: Androgen deprivation therapy
CAPRA: Cancer of the prostate risk assessment
EAU: European Association of Urology
ISUP: International Society of Urological Pathology
MSKCC: Memorial Sloan-Kettering Cancer Center
NCCN: National Comprehensive Cancer Network
PCa: Prostate cancer
PSA: Prostate-specific antigen
